# Community drug retail outlet staff’s knowledge, attitudes and practices towards non-prescription antibiotics use and antibiotic resistance in the Amhara region, Ethiopia with a focus on non-urban towns

**DOI:** 10.1186/s13756-022-01102-1

**Published:** 2022-04-29

**Authors:** Sewunet Admasu Belachew, Lisa Hall, Linda A. Selvey

**Affiliations:** 1 School of Public Health, The University of Queensland, 288 Herston Road, Herston, QLD 4006 Australia; 2 School of Pharmacy, Faculty of Medicine and Health Sciences, University of Gondar, Gondar, Ethiopia

**Keywords:** Antibiotic dispensing, Community pharmacy, Ethiopia, Knowledge, Non-urban

## Abstract

**Introduction:**

Some evidence suggests that knowledge and attitudes towards rational antibiotic use influences dispensing practice in community drug retail outlets. However, there is limited evidence in resource limited countries, including Ethiopia. We aimed to assess the knowledge and attitudes surrounding antibiotic use or supply and antibiotic resistance, and the non-prescribed antibiotic dispensing practices in community drug retail outlets in non-urban Ethiopia.

**Methods:**

We conducted a cross-sectional survey of community drug retail outlet staff in the Amhara region, Ethiopia with a focus on non-urban towns. An expert validated self-administered questionnaire was used. Following exploratory factor analysis and best items selection, we summarised our findings and assessed factors associated with non-prescribed antibiotic dispensing. The data were analysed using Stata Statistical Software version 17. *P*-values < 0.05 were considered significant.

**Results:**

A total of 276 participants from 270 drug outlets completed the questionnaire. The participants median age was 30 (Interquartile range (IQR) = 25–35) years and 79.7% were pharmacy assistants. The majority demonstrated good levels of knowledge about antibiotic use or supply and antibiotic resistance (77.9% and 76% of the participants responded correctly to more than half of the items, respectively). We identified four attitude domains: the role of antibiotics in recovering from diseases regardless of their cause (median score = 2 (IQR = 2–4), beliefs inconsistent with good practice); professional competency to supply non-prescribed antibiotics, and the non-prescribed antibiotics supply (median score for each domain = 4 (IQR = 4–5), attitudes consistent with good practice); and positive attitudes towards actions to prevent antibiotic resistance and promote appropriate antibiotic use (median score = 4 (IQR = 4–5). Fifty eight percent of the participants reported that they had dispensed antibiotics without a prescription. Participants who did not perceive that they were competent to supply non-prescribed antibiotics (adjusted odds ratio = 0.86, 95% confidence interval = 0.78–0.93) were less likely to report non-prescribed antibiotics dispensing.

**Conclusion:**

While most of the participants had appropriate knowledge about and attitudes to antibiotic use and antibiotic resistance, basic knowledge and attitude gaps remain. Despite Ethiopia’s regulatory restrictions, the non-prescribed antibiotic provision continues to be a common practice. Our study highlights the need for multifaceted interventions that may include a strict regulatory system, staff training and public education.

**Supplementary Information:**

The online version contains supplementary material available at 10.1186/s13756-022-01102-1.

## Introduction

Antibiotics are the most frequently used medicines, with a 46% increase in consumption worldwide after 2000 by the year 2018 [1]. The World Health Organization (WHO) has classified antibiotics into three categories according to their recommended use: access, watch and reserve, with the latter two categories having a higher potential for developing antibiotic resistance (ABR) [2]. Between 2000 and 2015, there has been a marked increase in the consumption of ‘watch’ antibiotics globally, particularly in low- and middle-income countries (LMICs) [3]. The increase in the consumption of antibiotics is driving an increase in ABR [3, 4], and markedly increases with mis or overuse [5–9]. It has been estimated that around 700,000 people lose their lives globally each year due to ABR infections [10], with the potential to increase to 10 million deaths by 2050 [10, 11].

Self-treatment with antibiotics has been flagged as one of the most common and obvious contributing factors to antibiotic mis- or overuse. This can result not only in individual harm (e.g., worsening of a health situation due to incorrect self-diagnosis and the wrong choice of therapy, avoidable adverse reactions and wastage of resources or increased healthcare cost), but also increases antibiotic consumption overall [12, 13].

Community drug retail outlets (CDROs) (pharmacies, drug stores/shops, rural drug vendors, and Accredited Drug Dispensing Outlets that sell drugs or medicines) are reported as the principal channel to access antibiotics for self-medication, particularly in LMIC settings including Ethiopia [12, 14, 15]. Despite the legal requirement to dispense antibiotics only with prescription, the non-prescription dispensing of antibiotics continues to be a common practice in LMICs [5]. Two systematic reviews and meta-analyses, one of which was conducted around the world [16] and the other that was conducted in Sub-Saharan Africa [17] reported that over 60% of CDROs dispensed antibiotics without a prescription. In this regard, several factors including CDRO staff’s knowledge of and attitudes towards antibiotic use or supply and ABR may explain the dispensing of antibiotics without prescription [18–25].

In Ethiopia, CDROs include pharmacies; which are medicine shops having the mandate to hold a range of medicines; drug stores are medicine shops with a more limited range of medications, and rural drug vendors, usually found in rural areas with an even more limited range of medications. Unlike Accredited Drug Dispensing Outlets in Tanzania which can be supervised by a person who attended a five-week training course [26, 27], the CDROs of Ethiopia are required to be supervised by a pharmacist or pharmacy assistant.

The CDRO dispensing practice in Ethiopia is regulated by the Ethiopian Food and Drug Authority (EFDA). EFDA released a directive in 2019, which clearly states that any dispenser should not dispense prescription only medication without a prescription [28]. The directive also noted that those who contravene the rules will receive sanctions ranging from verbal or written warnings to suspension or revoking of their license [28]. Although the authority prohibits the dispensing of antibiotics without prescription, the non-prescribed antibiotic dispensing was reported by different studies in Ethiopia showing that the regulations are not strongly enforced [29–31]. The non-prescribed antibiotic dispensing is believed to be higher in rural areas of Ethiopia as the regulatory system may be absent or less stringent. A recent nationwide study in China found that non-prescribed antibiotics dispensing was more prevalent in rural areas [32]. This suggests that interventional strategies and further research targeting rural areas should be a priority. However, we have not found any previous multicentre studies conducted in rural areas of Ethiopia that confirmed our hypothesis.

This study aimed to assess CDRO staff’s knowledge of and attitudes towards antibiotics use or supply and ABR, and the non-prescribed antibiotic dispensing practices as well as to explore the relationship between knowledge and attitudes and dispensing practice with a focus on non-urban CDROs in the Amhara region, Ethiopia. We anticipate that the evidence generated by this study will inform the regulatory policies and targeted interventions aimed at improving antibiotic use and dispensing behaviour in rural areas in Ethiopia.

## Methods

### Study setting and design

The survey was conducted in the Amhara Regional State, Ethiopia. The Amhara Regional State, is one of the largest and most geographically diverse regions, and is the second most populous region [33]. The region is divided into eleven zones for administrative purposes. Each zone is composed of several woredas; woredas are the third-level of the administrative division of Ethiopia after zones and regions [34].

A cross-sectional study using a self-administered questionnaire was conducted among CDRO staff in pharmacies, drug stores and rural drug vendors located in the two large provinces or divisions (Gondar and Gojam) in the Amhara region. Gondar and Gojam, which are composed of several zones and woredas, were purposively selected because they are the largest provinces or divisions in the Amhara region both geographically and in population size and have many and diverse CDROs in addition to their convenience for the study.

### Study sampling

In Gondar and Gojam excluding the two main cities (Gondar and Bahir-dar), there are 443 CDROs. All CDROs in Gondar except Gondar city, and all CDROs in Gojam (except the city of Bahir-dar) in woredas or towns near to Gondar were invited to participate. Information about the location and number of CDROs available in each woreda or town was obtained from the district health office. The survey covered 45 woredas or towns in total; 23 of which were in Gondar.

### Survey instrument

The structured questionnaire was developed from those used in previous studies developed from expert validated research instruments [25, 35–37]. The face and content validity of the draft survey was assessed by four experts from Australia and Ethiopia (senior pharmacologist, clinical pharmacist, clinician, pharmacist /behavioural science researcher). Questions were modified, added, or deleted to reduce ambiguity.

The questionnaire was pretested in ten purposively selected CDROs in Gondar city to obtain feedback about the questionnaire clarity, comprehension, and time needed to complete all the items. The survey tool, the participant information sheet and consent form were first developed in English, then translated to the local language (Amharic) and back translated to English.

The final version has four sections (Additional file 1): characteristics of CDROs and their CDRO staff, knowledge about antibiotic use or supply and ABR (33 questions); attitudes towards antibiotic use (29 questions); and non-prescribed antibiotic dispensing practice.

### Data collection

All active CDROs in the study towns were visited by trained data collectors who were either pharmacy postgraduate students or recent pharmacy graduates. The field supervisor closely monitored the data collection process in-person.

The questionnaire was provided to consenting participants in their preferred language (Amharic or English). CDRO staff either completed the questionnaire on the spot or arranged for survey collection at a suitable time and day.

### Ethical considerations

The study was approved by the University of Queensland Human Research Ethics Committee (approval number 2020002195), Australia and University of Gondar Institutional Ethical Review Board, Ethiopia (approval number V/P/RCS/05/412/2020). Written consent was obtained from each participant.

### Item response coding

#### Knowledge section

The participants response to each item was coded as 0 = no, 1 = unsure, and 2 = yes. In case of negatively quoted questions (where ‘no’ was the correct answer), reverse scoring was used.

#### Attitudes section

These questions used a 5-point Likert scale and each item was coded as 1 = strongly disagree, 2 = disagree, 3 = neither agree nor disagree, 4 = agree, and 5 = strongly agree. In case of negatively quoted questions where ‘disagree ‘was the appropriate response, reverse scoring was used.

#### Practice section

The level of practice assessed on a 5-point Likert scales as 0 = never, 1 = some of the time, 2 = half of the time, 3 = most of the time, 4 = always. Responses other than never were scored as dispensed. The participants response to the item “I dispense antibiotic/s without prescription” was used to score as dispensed or not.

### Data analysis

The data were analysed using STATA (StataCorp. 2021.Stata Statistical Software: Release 17. College Station, TX: StataCorp LLC). The data were analysed using descriptive statistics including frequencies and median (inter-quartile range (IQR). The Chi-square test was used to assess the significance of differences in frequencies of practice between characteristics. Correlation coefficients were estimated between the items measuring knowledge and items that were correlated (correlation coefficient ≥ 0.3) and conceptually related items were grouped. The item with the higher correlation coefficient with the other group items was selected as the ‘best items’ that belong to the group; as rule of thumb, if the coefficient was less than 0.3, the item was dropped from the scale [38]. Of the initial 33 items, 18 items were selected as best items measuring knowledge about antibiotics use or supply and ABR.

Exploratory factor analysis (EFA), was used to reduce the attitude items into meaningful factors explaining most of the total variances for the 29 items evaluating attitudes. Before performing the factor analysis, Kaiser–Meyer–Olkin (KMO) test for sampling adequacy and Bartlett’s test of sphericity were used to measure data suitability for factor analysis. KMO of 0.868 and Bartlett’s test of sphericity significance at *P* < 0.001, suggested that the dataset was suitable. Factors were extracted using principal axis factoring and retained when the eigenvalue was > 1 [38]. Oblimin rotation was used to assess which variables belongs most strongly to each factor. An item was said to load on a factor when the coefficient was ≥ 0.30, otherwise it was dropped from the scale [38]. The weighted factor-based score was created considering the actual reported value and factor loading value [38]. All 29 attitude items were included in the initial EFA. Five items were dropped from the final factor solution as they had a factor loading of < 0.30 or low commonalities [38]. The EFA retained four factors with eigenvalue greater than 1, explaining 98% of the total variance in the observed variables.

The reliability of each construct was computed using Cronbach’s alpha. The Cronbach’s alpha for items assessing knowledge about antibiotics use or supply domain was 0.801, indicating a good level of internal consistency, and was 0.648 for items assessing knowledge about ABR, indicating an acceptable level of internal consistency. The Cronbach’s alpha for items assessing attitudes towards the role of antibiotics in recovering from health conditions regardless of the cause was 0.944; was 0.856 for attitudes towards non-prescription dispensing of antibiotics; was 0.879 for attitudes towards professional competency to supply non-prescribed antibiotics; and was 0.790 for attitudes regarding the actions to prevent ABR and/or promote appropriate use.

In addition, percentages of participants’ response for each item measuring knowledge, attitudes and practices were calculated and presented. The percentages of response were also calculated for each group of items or domain measuring knowledge and attitudes to better interpret the findings.

Binary logistic regression was used to assess the association between variables and self-reported non-prescribed antibiotics dispensing practice. Multicollinearity and linearity of relationship between the continuous independent variables and the logit transformation of dependent variable was checked using the Box-Tidwell transformation test. The variables with a *P*-value < 0.20 in the univariable analysis were considered potentially confounding. Reverse stepwise regression was employed, and the covariates in the multivariable model were checked as they were eliminated for potential undue influence on the model stability, and model fit statistics (Akaike’s Information Criterion [AIC] and Schwarz’s Bayesian Information Criteria [BIC]). The results were expressed as odds ratios (OR) and 95% confidence intervals (CIs).

## Results

### Characteristics of the community drug retail outlets (CDROs) and the participating staff

Of the 318 staff within 312 CDROs who were approached, 276 from 270 CDROs participated in the survey (response rate of 86.8%). Two staff members participated from six CDROs.

The median age of the participants was 30 (IQR = 25–35) years, and almost half were aged 20–29 years (49.6%). Most of the participants were pharmacy assistants (79.7%) (Table 1). Drug stores were the commonest 204/270 (75.6%).

**Table 1:** Characteristics of the participating staff (N = 276)

Participant characteristics	Overall N = 276	Dispensed N = 160 (58%)	Never dispensed N = 116 (42%)	*X*^2^ *P*-value
Gender				0.457
Male	169 (61.2)	95 (56.2)	74 (43.8)	
Female	107 (38.8)	65 (60.7)	42 (39.3)	
Employment type				0.396
CDRO owner	144 (52.2)	80 (55.6)	64 (44.4)	
Employee	132 (47.8)	80 (60.6)	52 (39.4)	
Employment status				0.196
Full time	231 (83.7)	130 (56.3)	101 (43.7)	
Part time	45 (16.3)	30 (66.7)	15 (33.3)	
Current registration/licensing status of participants on duty				0.691
Registered	245 (88.8)	141 (57.6)	104 (42.4)	
Not registered	31 (11.2)	19 (61.3)	12 (38.7)	
Trained about medication use/dispensing practice in the last 3 years				0.579
Yes	37 (13.4)	23 (62.2)	14 (37.8)	
No	239 (86.6)	137 (57.3)	102 (42.7)	
Educational status				0.028*
Pharmacy assistant	220 (79.7)	131 (59.5)	89 (40.5)	
Pharmacist	47 (17)	21 (44.7)	26 (55.3)	
Non-pharmacy professional	9 (3.3)	8 (88.9)	1 (11.1)	
Age in years				0.815
20–29	137 (49.6)	82 (59.9)	55 (40.1)	
30–39	100 (36.2)	58 (58)	42(42)	
40–49	20(7.2)	10 (50)	10 (50)	
≥ 50	19 (6.9)	10 (52.6)	9 (47.4)	
Work experience in CDRO (in years)				0.482
≤ 5	143 (51.8)	87 (61.3)	55 (38.7)	
5–10	86 (31.2)	47 (54.7)	39 (45.3)	
> 10	47 (17)	25 (53.2)	22 (46.8)	
Type of workplace				0.047*
Pharmacy	59 (21.4)	27 (45.8)	32 (54.2)	
Drug store	207 (75)	125 (60.4)	82 (39.6)	
Rural drug vendor	10 (3.6)	8 (80)	2 (20)	
Workplace structure				0.308
Chained CDRO	48 (17.4)	23 (51.1)	22 (48.9)	
Independent CDRO	228 (82.6)	137 (59.3)	94 (40.7)	
Workplace distance from health facilities				0.009*
Within 1 km	217 (78.6)	117 (53.9)	100 (46.1)	
Greater than 1 km	59 (21.4)	43 (72.9)	16 (27.1)	
Workplace distance from the centre of the town				0.147
Within 1 km	235 (85.1)	132 (56.2)	103 (43.8)	
Greater than 1 km	41 (14.9)	28 (68.3)	13 (31.7)	

^*^Significant at *P* < 0.05

Over half (58%) of participants reported that they have dispensed antibiotics without prescription at least some of the time in the past. A significantly higher proportion of participants working in CDROs greater than 1 km of health facilities 43/59 (72.9%) reported dispensing non-prescribed antibiotics compared to those who work in CDROs within 1 km of health facilities 117/217 (53.9%), (*x*^2^ (1, N = 276) = 6.847, *P* = 0.009) (Table 1).

### Type of non-prescribed antibiotics most dispensed in the CDROs

One hundred and fifty-three participants named the type of antibiotics most frequently dispensed without prescription in their CDROs. Nearly all 141/153 (92.2%) reported supplying amoxicillin, 68/153 (44.4%) ciprofloxacin and 64/153 (41.8%) cloxacillin without prescription in their CDROs (Additional file 2).

### Most common symptoms or disease conditions for which antibiotics were dispensed without prescription

**Fig. 1:**
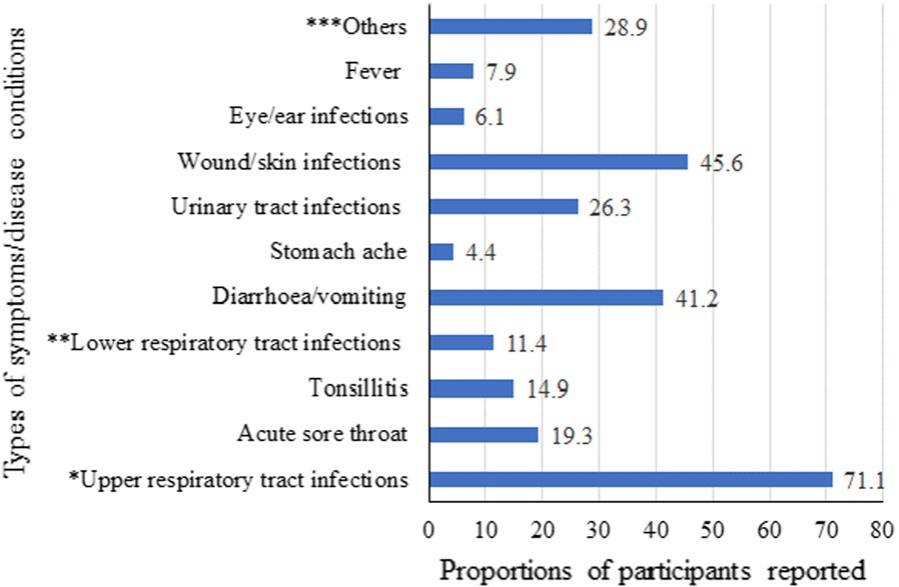
Most common symptoms/disease conditions for which non-prescribed antibiotics were dispensed as reported by the 114 participants. ***Others includes parasitic and bacterial infections, toothache, peptic ulcer disease, womb infection, arthritis, headache, and various infections. *Upper respiratory tract infections (URTIs) includes participants’ response noted as URTIs in general and/or cough and/or common cold. **Lower respiratory tract infection (LRTI) includes pneumonia

Of the participants who dispensed antibiotics without prescription, 114 reported the main symptoms or disease conditions for which they dispensed antibiotics. The most common condition reported was upper respiratory tract infections (URTIs) 81/114 (71.1%) (Fig. 1).

### Knowledge

The items were categorised into two domains according to their conceptual correspondence (Table 2): *Knowledge about antibiotics use or supply and knowledge about ABR*:

**Table 2:** Frequency of correct, incorrect, and unsure responses to knowledge items (N = 276)

Items	Correct n (%)	Incorrect n (%)	Unsure n (%)
*Knowledge about antibiotics use or supply*			
An antibiotic is a medicine used to kill or inhibit the growth of bacteria, fungus, virus, and parasites	153 (55.4)	117 (42.4)	6 (2.2)
Diphenhydramine is an antibiotic used in treating upper respiratory tract infections	170 (61.6)	99 (35.9)	7 (2.5)
Diseases with viral causes can be treated with antibiotics	179 (64.8)	88 (31.9)	9 (3.2)
An antibiotic is any agent used to kill or inhibit the growth of bacteria	259 (93.8)	13 (4.7)	4 (1.5)
If taken too often without a clear indication, antibiotics are less likely to work in the future	257 (93.1)	16 (5.8)	3 (1.1)
Antibiotics can be used as a preventive measure to fight against future microbial (i.e., fungus, bacteria, virus, and parasite) attacks	238 (86.2)	36 (13.1)	2 (0.7)
Keeping leftover antibiotics from a previous course to use next time for the same infection is a good practice	250 (90.6)	25 (9)	1 (0.4)
Patients can stop taking antibiotics when their symptoms improve before completing their prescribed course of therapy	212 (76.8)	54 (19.6)	10 (3.6)
Acute sore throat can be treated with antibiotics irrespective of the cause	198 (71.7)	72 (26.1)	6 (2.2)
Fever can be treated directly with antibiotics	229 (83)	42 (15.2)	5 (1.8)
Common cold and cough should always be treated with antibiotics	216 (78.3)	54 (19.5)	6 (2.2)
Wound infection can be treated with antibiotics irrespective of the cause	143 (51.8)	130 (47.1)	3 (1.1)
Acute diarrhoea can be treated with antibiotics irrespective of the cause	178 (64.5)	91 (33)	7 (2.5)
Urinary tract infections can be treated with antibiotics irrespective of the cause	143 (51.8)	125 (45.3)	8 (2.9)
*Knowledge about antibiotic resistance*			
Inappropriate use of antibiotics increases the emergence of bacterial resistance to antibiotics	224 (81.2)	46 (16.6)	6 (2.2)
Dispensing antibiotics without a prescription will lead to development of antibiotic resistance	214 (77.5)	54 (19.6)	8 (2.9)
In complete antibiotic course is one of the causes of antibiotic resistance	228 (82.6)	43 (15.6)	5 (1.8)
Clients’ self-medication with antibiotics contributes to the development of antibiotic resistance	206 (74.6)	58 (21)	12 (4.4)

*Knowledge about antibiotics use or supply.* The percentages of participants who correctly responded to knowledge questions regarding antibiotics use or supply ranged from 51.8% for two questions assessing ‘knowledge about antibiotic use for wound infection and urinary tract infection regardless of the causes’ to 93.8% for the ‘knowledge about antibiotic use as an agent to kill or inhibit the growth of bacteria’. However, nearly half of the participants were unsure or agreed that ‘without taking the cause into account antibiotics can be used for urinary tract infections (48.2%), and wound infections (48.2%)’. Most of the participants (77.9%) responded correctly to more than half of the items, and 64.5% answered correctly to at least 10 of the 14 items.

*Knowledge about ABR*. The percentages of participants who gave the correct answer to questions assessing knowledge about ABR ranged from 74.6% (assessing knowledge about the role of clients’ self-medication with antibiotics in contributing to the development of ABR) to 82.6% (assessing knowledge about the relationship between incomplete antibiotic course and the development of ABR). Seventy six percent of the participants responded correctly to more than half of the items, 54.7% responded correctly to all items and 4.7% did not respond correctly to any items.

### Attitudes

The items were categorised in to four domains (Table 3):

**Table 3:** The level of participants’ agreement in each attitude statement (N = 276)

Items	Strongly disagree n (%)	Disagree n (%)	Never agree nor disagree n (%)	Agree n (%)	Strongly agree n (%)	Factor loading			
						Factor 1	Factor 2	Factor 3	Factor 4
*Factor 1: Attitudes towards the roles of antibiotics in recovering from health conditions irrespective of the causes*									
I believe antibiotics may have a role in recovering from the following health conditions, irrespective of their cause,									
Fever	75 (27.2)	79 (28.6)	7 (2.5)	93 (33.7)	22 (8)	0.7562			
Common cold and Cough	65 (23.5)	67 (24.3)	13 (4.7)	104 (37.7)	27 (9.8)	0.8005			
Acute sore throat	57 (20.6)	51 (18.5)	11 (4)	123 (44.6)	34 (12.3)	0.8787			
Acute diarrhoea	61 (22)	57 (20.7)	9 (3.3)	118 (42.8)	31 (11.2)	0.8709			
Wound infection	48 (17.4)	38 (13.8)	10 (3.6)	133 (48.2)	47 (17)	0.9052			
Uncomplicated urinary tract infection	50 (18.1)	41 (14.9)	11 (4)	137 (49.6)	37 (13.4)	0.9393			
*Factor 2: Attitudes towards non-prescription dispensing of antibiotics*									
The CDRO staff should dispense antibiotics without prescription because refusing to dispense antibiotics without a prescription will negatively affect the CDRO’s sales and profits	121 (43.8)	108 (39.1)	15 (5.5)	19 (6.9)	13 (4.7)		0.6902		
The education I acquired or the knowledge I have about antibiotics is adequate to allow me to dispense antibiotics without a prescription	92 (33.3)	110 (39.9)	12 (4.3)	45 (16.3)	17 (6.2)		0.7629		
The CDRO staff should dispense antibiotics without prescription because refusing clients’ request to dispense antibiotics without prescription may cause client dissatisfaction with the CDRO service	103 (37.3)	110 (39.9)	16 (5.8)	32 (11.6)	15 (5.4)		0.8487		
The CDRO staff should dispense antibiotics without prescription as clients may easily obtain a prescription from their doctor	98 (35.5)	129 (46.7)	8 (3)	34 (12.3)	7 (2.5)		0.5354		
As the pressure from the CDRO owner to increase sales and profits is high, the CDRO staff should dispense antibiotics without a prescription	112 (40.6)	115 (41.7)	17 (6.1)	23 (8.3)	9 (3.3)		0.6607		
The CDRO staff should dispense antibiotics without prescription as they will get it easily from another retail outlet anyway	98 (35.5)	105 (38)	20 (7.3)	35 (12.7)	18 (6.5)		0.3999		
If a patient with a bacterial infection visits a CDRO, the CDRO staff should dispense antibiotics without asking for a prescription	106 (38.4)	118 (42.8)	14 (5)	33 (12)	5 (1.8)		0.4146		
If CDRO staff know that clients have neither the time nor the finance to visit a doctor, they should dispense antibiotics without the prescription	110 (39.8)	107 (38.8)	21 (7.6)	30 (10.9)	8 (2.9)		0.4122		
*Factor 3: Attitudes towards professional competency to supply non-prescribed antibiotics*									
Dispensing antibiotics without a prescription is not a problem because,									
I believe that antibiotics are safe medicines	138 (50)	102 (37)	9 (3.2)	21 (7.6)	6 (2.2)			0.7562	
I believe that antibiotics have no or few side effects	118 (42.8)	114 (41.3)	7 (2.5)	28 (10.1)	9 (3.3)			0.7774	
I have the ability to assess the patients’ need for antibiotics	105 (38)	113 (40.9)	18 (6.5)	31 (11.3)	9 (3.3)			0.8216	
I know whether to dispense antibiotics to the patient or refer them to a doctor	94 (34.1)	99 (35.9)	12 (4.3)	47 (17)	24 (8.7)			0.7623	
I can properly counsel patients on appropriate use of antibiotics	94 (34.1)	95 (34.4)	16 (5.8)	54 (19.5)	17 (6.2)			0.7728	
*Factor 4: Attitudes regarding the actions to prevent antibiotic resistance and/or promote appropriate use*									
Clients should stop self-medicating with antibiotics to prevent the occurrence of antibiotic resistance	39 (14.1)	26 (9.4)	13 (4.7)	104 (37.7)	94 (34.1)				0.5545
The CDRO staff should not dispense antibiotics without a prescription because provision of antibiotics without prescription leads to the development of resistance	45 (16.3)	33 (12)	19 (6.9)	97 (35.1)	82 (29.7)				0.4158
Dispensing antibiotics without a prescription should be more closely controlled by the authorities	29 (10.5)	20 (7.2)	22 (8)	110 (39.9)	95 (34.4)				0.7414
A CDRO staff should encourage patients to consult a physician and get a prescription before visiting the CDRO	33 (12)	18 (6.5)	8 (2.9)	89 (32.2)	128 (46.4)				0.7632
Promoting appropriate use of antibiotics is a shared responsibility of healthcare professionals, patients, and policy makers	29 (10.5)	23 (8.4)	10 (3.6)	95 (34.4)	119 (43.1)				0.7588
*Cronbach’s alpha*						0.944	0.856	0.879	0.790

Five items were dropped due to low factor loading was (< 0.3) or low commonalities. All factors had increasing scale ranging between 1 = strongly disagree to 5 strongly agree. Reverse coding was instituted for negative statements

*Attitudes towards the role of antibiotics in recovering from health conditions regardless of the cause*. The overall median score for this domain was two (IQR = 2–4, out of 1–5 increasing Likert scale), indicating inappropriate attitudes by agreeing with the statement that without taking the cause into account, antibiotics may have a role in recovering from health conditions including acute sore throat (56.9%), acute diarrhoea (54%), common cold and cough (47.5%) and wound infection (65.2%).

*Attitudes towards non-prescription dispensing of antibiotics*. The majority of the participants disagreed or strongly disagreed with the statements that ‘antibiotics should be dispensed without prescription because: clients may easily obtain a prescription from their doctor (82.2%); the pressure from the CDRO owner to increase sales and profits is high (82.3%), and refusal will affect the CDROs’ sales profit (82.9%). Overall, the median score for the items measuring attitudes towards non-prescription dispensing of antibiotics was four (IQR = 4–5, out of the 1–5 increasing Likert scale), indicating better attitudes of participants in relation to discouraging the non-prescription antibiotic supply.

*Attitudes towards professional competency to supply non-prescribed antibiotics.* The majority of participants disagreed or strongly disagreed with the statements that antibiotics can be dispensed without prescription because: antibiotics are safe medicines (87%); have no or few side effects (84.1%). The median score for the six items measuring attitudes towards professional competency to supply non-prescribed antibiotics was 4 (IQR = 4–5, out of the 1–5 increasing Likert scale), indicating that the participants did not perceive that they were competent to provide antibiotics without prescription.

*Attitudes regarding the actions to prevent ABR and/or promote appropriate use*. Most of the participants agreed or strongly agreed with the statement that prohibiting clients’ self-medication with antibiotics prevents the occurrence of ABR (71.8%), and that ensuring proper use of antibiotics is a shared responsibility of healthcare professionals, patients, and policy makers (77.5%). The median score for each of the 5 items in this domain was 4 (IQR = 4–5, out of the 1–5 increasing Likert scale), indicating appropriate attitudes towards the actions to prevent ABR and/or promote appropriate use.

### Non-prescription antibiotic dispensing practice

Of the 160 participants who reported dispensing antibiotics without prescription at least some of the time, greater than half 92/160 (57.5%) reported that they have dispensed antibiotics without a prescription up on direct request from a client. Most (74%, 118/160) of these participants reported that they had provided antibiotics without prescription for common cold and cough conditions in the last week (Table 4).

**Table 4:** Findings of participants’ frequency of practice for each statement (N = 276)

Items	Never n (%)	Some of the time n (%)	Half of the time n (%)	Most of the time n (%)	Always n (%)
I dispense antibiotic/s without prescription	116 (42)	106 (38.4)	35 (12.7)	8 (2.9)	11 (4)
I dispense antibiotics without a prescription up on direct request from a client	184 (66.7)	57 (20.7)	16 (5.8)	7 (2.5)	12 (4.3)
For adult with bacterial infection, I provide antibiotics without asking for a prescription	200 (72.5)	47 (17)	16 (5.8)	2 (0.7)	11 (4)
I dispense antibiotics without prescription for a family member or someone I know well	187 (67.8)	57 (20.6)	17 (6.2)	6 (2.2)	9 (3.2)
For patients with minor ailments, I dispense antibiotics as an over-the-counter drug	157 (56.9)	76 (27.5)	29 (10.5)	6 (2.2)	8(2.9)
I dispense antibiotics without prescription for children with bacterial infection	201 (72.8)	47 (17)	15 (5.4)	4 (1.4)	9(3.3)
For a child with viral infection, I dispense antibiotics without prescription	236 (85.5)	22 (8)	8 (2.9)	3 (1.1)	7 (2.5)
I dispense antibiotics without a prescription for an adult with viral infection	231 (83.7)	25 (9.1)	10 (3.6)	2 (0.7)	8 (2.9)
Have you dispensed antibiotic/s without prescription for the following condition in the last week, in what level?					
Common cold or cough	158 (57.2)	71 (25.7)	35 (12.7)	6 (2.2)	6 (2.2)
Acute diarrhoea	177 (64.1)	49 (17.8)	36 (13)	8 (2.9)	6 (2.2)
Uncomplicated urinary tract infection	186 (67.4)	50 (18.1)	25 (9)	9 (3.3)	6 (2.2)
Wound infection	176 (63.8)	48 (17.4)	32 (11.6)	10 (3.6)	10 (3.6)
Acute sore throat	187 (67.8)	50 (18.1)	23 (8.3)	7 (2.5)	9 (3.3)
In what level, do you currently dispense antibiotic/s without prescription if you encounter a patient with,					
Common cold or cough	155 (56.1)	74 (26.8)	25 (9.1)	16 (5.8)	6 (2.2)
Acute diarrhoea	178 (64.5)	49 (17.8)	30 (10.9)	12 (4.3)	7 (2.5)
Uncomplicated urinary tract infection	179 (64.9)	55 (19.9)	25 (9.1)	9 (3.3)	8 (2.9)
Wound infection	168 (60.9)	47 (17)	41 (14.9)	10 (3.6)	10 (3.6)
Acute sore throat	186 (67.4)	38 (13.8)	30 (10.9)	12 (4.3)	10 (3.6)

### Factors associated with the practice of dispensing non-prescribed antibiotics at least some time in the past

Participants who did not agree with their professional competency to supply non-prescribed antibiotics were less likely to report dispensing antibiotics without prescription than those who feel competent (adjusted odds ratio (aOR) = 0.86, 95% confidence interval (CI) = 0.78–0.93). The univariable models showed a significant association between CDROs’ distance from health facilities (crude odds ratio (cOR) = 0.45, 95% CI = 0.24–0.85) and type of CDRO (cOR = 1.88, 95% CI = 1.05–3.35) and non-prescribed antibiotic dispensing. Other covariates considered in the initial model include knowledge score, employment type and status, licensing status, previous training, gender, workplace distance from the centre of the town, and workplace structure (Table 5).

**Table 5:** Factors associated with the non-prescribed antibiotics dispensing practice at least some time in the past

Variables	Non-prescribed antibiotics		Crude OR (95%CI)	Adjusted OR (95%CI)
	Dispensed (at least sometime) n (%)	Never dispensed n (%)		
Attitudes towards non-prescription dispensing of antibiotics	160 (58)	116 (42)	0.85 (0.79–0.91) *	0.93 (0.86–1.00)
Attitudes towards professional competency to supply non-prescribed antibiotics	160 (58)	116 (42)	0.81 (0.75–0.88) *	0.86 (0.78–0.93) *
Work experience	160 (58)	116 (42)	0.96 (0.93–0.99) *	0.97 (0.93–1.01)
Type of work place				
Non-pharmacy (drug store or rural drug vendor)	133 (83.1)	84 (72.4)	1.88 (1.05–3.35) *	1.65 (0.88–3.09)
Pharmacy	27 (16.9)	32 (27.6)	1 (ref)	1 (ref)
Work place distance from health facilities				
Within 1 km	117 (53.9)	100 (46.1)	0.45 (0.24–0.85) *	0.56 (0.28–1.12)
Greater than 1 km	43 (72.9)	16 (27.1)	1 (ref)	1 (ref)

^*^Significant at *P* < 0.05

## Discussion

Although there is a growing body of evidence suggesting that knowledge and attitudes of CDRO staff towards rational antibiotic use influences their dispensing practices, a dearth of evidence exists regarding CDRO staff’s views in non-urban CDROs, where the level of compliance to and enforcement of dispensing-related regulatory policies may be different from urban settings. This is the first multicentre study that explored the knowledge, attitudes, and self-reported practices of CDRO staff with a focus on non-urban areas of Ethiopia.

Most of the participants had satisfactory levels of knowledge about antibiotic use or supply, and ABR. This finding is consistent with other studies conducted in low- and middle-income countries [25, 35, 39–41]. We found that nearly half of the participants (44.6%) did not know or were unsure that antibiotics are effective only against bacteria by indicating agreement that antibiotics kill or inhibit the growth of fungus, virus, parasites, and bacteria. This knowledge could be partially explained by the fact that our respondents were predominantly pharmacy assistants (79.7%), with limited training on pharmacotherapy and pharmacology.

Nearly half of the participants agreed with the statements that antibiotics can be used to treat some conditions commonly treated by antibiotics without taking the cause into account. While there are clinical indications where antibiotics are initiated as an empirical therapy to target the most probable causative microorganisms (based on local susceptibility data, available scientific evidence, or expert opinion) [42], a blanket treatment of all minor ailments or conditions with antibiotics without taking possible or probable causes into account to rule out non-bacterial causes, may contribute towards ABR. The regulation requiring antibiotic access only with prescription may not be practicable to implement in many LMICs [43–45] such as Ethiopia because of a scarcity of healthcare facilities (the diagnostic capabilities are widely absent or limited to confirm the presence and type of microbe/s), and most of the population lives in rural areas [46]. In these situations, CDROs may serve as a primary healthcare facility and are a main source of medications [14, 15]. We suggest that CDRO staff be equipped with the necessary knowledge and skills for empirical treatment of common conditions with clear symptoms and/or signs (such as urinary tract infections and wound infections) based on the country’s treatment guidelines at least in rural or remote areas to improve appropriate antibiotic access.

Most participants said they were not competent to supply non-prescribed antibiotics and demonstrated an understanding about appropriate use or supply. Studies conducted in Tanzania, Pakistan, Sri Lanka and Zambia also reported that CDRO staff had appropriate attitudes towards antibiotics use or supply [40, 47, 48], and ABR [39]. In contrast, considerable number of participants agreed that regardless of the causes, antibiotics may have a role for recovering from acute sore throat, diarrhoea, wound infection, uncomplicated urinary infection, common cold and cough. Anecdotal evidence in the study area indicates that this might be partly associated to the existing trend of supplying antibiotics in addition to a standard non-antibiotic treatment with the intention to increase the likelihood of recovery from a certain illness.

Even though it is illegal to dispense antibiotics without prescription, a lot of participants reported doing so. Our finding confirms the common practice of supplying antibiotics without prescription in Sub-Saharan Africa as previously reported [17]. The proportion of participants dispensing without a prescription in our study was lower than in reported studies conducted in Thailand and Tanzania which indicated that 99.6% and 74% of the pharmacists agreed that they dispensed antibiotics without prescription, respectively [35, 40]. In Thailand, the higher proportion might be because the licensed pharmacists are legally allowed to dispense most antibiotics without prescription except few reserve antibiotics [49].

Our study also found that the distance of CDROs from health facilities may be associated with dispensing without a prescription as CDROs closer to health facilities reported less dispensing. This may be because clients who visit CDROs near to health facilities would be more likely to have prescription available. Absence of health facilities in nearby areas have also been reported by previous studies as a factor for increased non-prescription antibiotics supply by CDROs [50–52]. Improving access to health facilities could be one potential strategy to contain the non-prescribed antibiotic supply, particularly in rural areas.

Non-prescribed dispensing of ‘watch’ antibiotics (e.g., ciprofloxacin (44.4%), macrolides (5.9%), and cephalosporins (9.8%)) was reported in our study. This increases the risk of ABR and could also be a great challenge to achieving the WHO target of making at least 60% of the national level total antibiotic consumption to be in the access category by 2023 [2, 3]. Furthermore, almost all the antibiotics reported were broad spectrum antibiotics. This might be because of CDRO staff preference to suggest broad-spectrum antibiotics as they will allow a broader range of pathogens to be covered thereby enhancing the possibility of therapeutic success. However, the use of broad-spectrum antibiotics seriously disturbs the normal intestinal flora, enabling bacterial overgrowth with emergent resistant microorganisms [8, 12, 53–55].

Despite the participants generally had good knowledge and appropriate attitudes towards antibiotic use or supply and ABR, knowledge and attitude gaps remain. Educational training programmes have been reported to improve healthcare professionals’ knowledge and attitudes towards antimicrobial stewardship [56]. However, the reported dispensing of antibiotics without prescription may be more likely to be a consequence of overarching health system issues and the business-centred approaches of CDROs rather than being due to knowledge and attitude gaps. Hence, a multi-faceted integrated approach is required to ensure judicious use of antibiotic including enacting new or a stricter enforcement of laws prohibiting the non-prescribed antibiotics supply [57–60], CDRO staff training about the importance of antimicrobial stewardship [61], and public education campaigns about the importance of preserving antibiotics [62–64].

The following implementation strategies could be beneficial in addressing our recommendations or the situation: capacity building at the regulatory body level such as increasing human resource, which may increase the frequency of CDRO inspections and improve law enforcement. In addition, controlling CDROs’ potential illegal sources of antibiotics through monitoring, and regularly conducting ‘prescription’ audits at the CDROs may facilitate effective tracking of the antibiotic transactions and enhance enforcement of the regulations. Furthermore, using public gatherings such us holidays, social events, or open markets to educate public about the harms of antibiotic over use may reduce community demand for antibiotics. Moreover, given that high profit motive is a reason for the sale of antibiotics without prescription [18], government subsidisation of CDROs such as with small scale business loans or reducing the cost of some essential drugs, may partly address their commercial interest, while delivering services in the right way. In addition, drug stores should be targeted with antimicrobial stewardship interventions as they are the most prevalent CDROs in most parts of Ethiopia. The regulatory bodies should also randomly visit the CDROs and verify the qualifications of the dispensers, especially those who are working in remote areas.

### Strengths and limitations

This was a multi-centre study conducted in highly populated places with CDROs believed to have multiples sources of medications. Thus, the evidence generated through this study would be a crucial input for stakeholders in targeting interventions. The high response rate was also a strength of this study. Our study has some limitations. Social desirability bias in relation to sharing information regarding antibiotic dispensing practice may underestimate antibiotic dispensing without a prescription. To address this, this study will be followed by a simulated client study to observe their practice in their natural working environment. Potential recall bias may be another limitation of the study. Although the CDRO structure is believed to be similar throughout the Amhara region, generalisability of the result to the entire Amhara region needs caution as our sample was not random. To enable a broader and deeper understanding of the CDRO staff dispensing behaviour, our study highlights the need for qualitative research.

## Conclusion

The majority of the CDRO staff surveyed had appropriate knowledge about antibiotics use or supply and ABR. Most of the staff also had appropriate attitudes about discouraging non-prescribed antibiotics provision and towards the actions to prevent ABR and/or promote appropriate use. However, there are basic knowledge and attitude gaps that need improvement. Despite the prohibition of the non-prescribed supply of antibiotics in Ethiopia, 58% of CDRO participants reported dispensing antibiotics without prescription. Our study highlights the need for a multi-faceted integrated intervention including enacting new or stricter enforcement of laws governing antibiotic dispensing, CDRO staff training about the importance of antimicrobial stewardship, public education campaigns, and establishing rigorous practice surveillance system.

## Supplementary Information

**Additional file 1:** Data collection instrument.

**Additional file 2: Fig. S1.** The types of antibiotics most dispensed without prescription as reported by the 153 participants.

## Data Availability

All relevant data are included in the paper.

## Abbreviations

ABR: Antibiotic resistance
aOR: Adjusted odds ratio
CI: Confidence interval
CDROs: Community drug retail outlets
cOR: Crude odds ratio
LMICs: Low- and middle-income countries
STATA: Stata Statistical Software
URTI: Upper respiratory tract infection

## References

[1] Browne AJ, Chipeta MG, Haines-Woodhouse G, Kumaran EPA, Hamadani BHK, Zaraa S (2021). Global antibiotic consumption and usage in humans, 2000–18: a spatial modelling study. Lancet Planet Health.

[2] World Health Organization. Home AWaRe (adoptaware.org). 2019 WHO. Privacy Legal Notice.

[3] Klein EY, Milkowska-Shibata M, Tseng KK, Sharland M, Gandra S, Pulcini C (2021). Assessment of WHO antibiotic consumption and access targets in 76 countries, 2000–15: an analysis of pharmaceutical sales data. Lancet Infect Dis.

[4] Bell BG, Schellevis F, Stobberingh E, Goossens H, Pringle M (2014). A systematic review and meta-analysis of the effects of antibiotic consumption on antibiotic resistance. BMC Infect Dis.

[5] Ayukekbong JA, Ntemgwa M, Atabe AN (2017). The threat of antimicrobial resistance in developing countries: causes and control strategies. Antimicrob Resist Infect Control.

[6] Chokshi A, Sifri Z, Cennimo D, Horng H (2019). Global Contributors to Antibiotic Resistance. J Glob Infect Dis.

[7] Llor C, Bjerrum L (2014). Antimicrobial resistance: risk associated with antibiotic overuse and initiatives to reduce the problem. Ther Adv Drug Saf.

[8] Prestinaci F, Pezzotti P, Pantosti A (2015). Antimicrobial resistance: a global multifaceted phenomenon. Pathog Glob Health.

[9] Ventola CL (2015). The antibiotic resistance crisis: part 1: causes and threats. Pharm Ther.

[10] New report calls for urgent action to avert antimicrobial resistance crisis. World Health organisation. 29 April 2019. https://www.who.int/news/item/29-04-2019-new-report-calls-for-urgent-action-to-avert-antimicrobial-resistance-crisis.

[11] O’Neill J. Review on AMR. Antimicrobial resistance: tackling a crisis for the health and wealth of nations. December, 2014. 2017.

[12] Nepal G, Bhatta S (2018). Self-medication with antibiotics in WHO Southeast Asian Region: a systematic review. Cureus.

[13] Rather IA, Kim BC, Bajpai VK, Park YH (2017). Self-medication and antibiotic resistance: crisis, current challenges, and prevention. Saudi J Biol Sci.

[14] Ayalew MB (2017). Self-medication practice in Ethiopia: a systematic review. Patient Prefer Adherence.

[15] Ocan M, Obuku EA, Bwanga F, Akena D, Richard S, Ogwal-Okeng J (2015). Household antimicrobial self-medication: a systematic review and meta-analysis of the burden, risk factors and outcomes in developing countries. BMC Public Health.

[16] Auta A, Hadi MA, Oga E, Adewuyi EO, Abdu-Aguye SN, Adeloye D (2019). Global access to antibiotics without prescription in community pharmacies: a systematic review and meta-analysis. J Infect.

[17] Belachew SA, Hall L, Selvey LA (2021). Non-prescription dispensing of antibiotic agents among community drug retail outlets in Sub-Saharan African countries: a systematic review and meta-analysis. Antimicrob Resist Infect Control.

[18] Belachew SA, Hall L, Erku DA, Selevy LA (2021). No prescription? No problem: drivers of non-prescribed sale of antibiotics among community drug retail outlets in low- and middle-income countries: a systematic review of qualitative studies. BMC Public Health.

[19] Erku DA, Mekuria AB, Belachew SA (2017). Inappropriate use of antibiotics among communities of Gondar town, Ethiopia: a threat to the development of antimicrobial resistance. Antimicrob Resist Infect Control.

[20] Lin L, Sun R, Yao T, Zhou X, Harbarth S (2020). Factors influencing inappropriate use of antibiotics in outpatient and community settings in China: a mixed-methods systematic review. BMJ Glob Health.

[21] Nahar P, Unicomb L, Lucas PJ, Uddin MR, Islam MA, Nizame FA (2020). What contributes to inappropriate antibiotic dispensing among qualified and unqualified healthcare providers in Bangladesh? A qualitative study. BMC Health Serv Res.

[22] Roque F, Soares S, Breitenfeld L, Figueiras A, Herdeiro MT (2015). Influence of community pharmacists׳ attitudes on antibiotic dispensing behavior: a cross-sectional study in Portugal. Clin Ther.

[23] Servia-Dopazo M, Figueiras A (2018). Determinants of antibiotic dispensing without prescription: a systematic review. J Antimicrob Chemother.

[24] Vazquez-Lago J, Gonzalez-Gonzalez C, Zapata-Cachafeiro M, Lopez-Vazquez P, Taracido M, López A (2017). Knowledge, attitudes, perceptions and habits towards antibiotics dispensed without medical prescription: a qualitative study of Spanish pharmacists. BMJ Open.

[25] Zawahir S, Lekamwasam S, Aslani P (2019). A cross-sectional national survey of community pharmacy staff: knowledge and antibiotic provision. PLoS ONE.

[26] Horumpende PG, Sonda TB, van Zwetselaar M, Antony ML, Tenu FF, Mwanziva CE (2018). Prescription and non-prescription antibiotic dispensing practices in part I and part II pharmacies in Moshi Municipality, Kilimanjaro Region in Tanzania: a simulated clients approach. PLoS ONE.

[27] Ndaki PM, Mushi MF, Mwanga JR, Konje ET, Ntinginya NE, Mmbaga BT (2021). Dispensing antibiotics without prescription at community pharmacies and accredited drug dispensing outlets in tanzania: a cross-sectional study. Antibiotics.

[28] Ethiopian Food and Drug Authority, Rational Medicine Use Control Directives, 2019. http://www.fmhaca.gov.et.

[29] Erku DA, Aberra SY (2018). Non-prescribed sale of antibiotics for acute childhood diarrhea and upper respiratory tract infection in community pharmacies: a 2 phase mixed-methods study. Antimicrob Resist Infect Control.

[30] Erku DA, Mekuria AB, Surur AS, Gebresillassie BM (2016). Extent of dispensing prescription-only medications without a prescription in community drug retail outlets in Addis Ababa, Ethiopia: a simulated-patient study. Drug Healthc Patient Saf.

[31] Koji EM, Gebretekle GB, Tekle TA (2019). Practice of over-the-counter dispensary of antibiotics for childhood illnesses in Addis Ababa, Ethiopia: a simulated patient encounter study. Antimicrob Resist Infect Control.

[32] Chang J, Xu S, Zhu S, Li Z, Yu J, Zhang Y (2019). Assessment of non-prescription antibiotic dispensing at community pharmacies in China with simulated clients: a mixed cross-sectional and longitudinal study. Lancet Infect Dis.

[33] World population review (2021). Based on projections of the latest United Nations data. https://worldpopulationreview.com/countries/ethiopia-population. Accessed 8 Oct 2021.

[34] Federal Ministry of Health, Health Sector Transformation Plan. HSTP 2015/16-2019/20. August; 2015.

[35] Siltrakool B. Assessment of community pharmacists’ knowledge, attitude and practice regarding non-prescription antimicrobial use and resistance in Thailand. 2018.

[36] Hadi MA, Karami NA, Al-Muwalid AS, Al-Otabi A, Al-Subahi E, Bamomen A (2016). Community pharmacists' knowledge, attitude, and practices towards dispensing antibiotics without prescription (DAwP): a cross-sectional survey in Makkah Province, Saudi Arabia. Int J Infect Dis.

[37] Zawahir MS. Pharmacists’ provision and public’s use of antibiotics for common infections in Sri Lanka (Doctoral dissertation); 2019.

[38] De Vaus DA (2014). Surveys in Social Research.

[39] Mudenda S, Hankombo M, Saleem Z, Sadiq MJ, Banda M, Munkombwe D et al. Knowledge, Attitude, and Practices of Community Pharmacists on Antibiotic Resistance and Antimicrobial Stewardship in Lusaka, Zambia. medRxiv. 2020.

[40] Poyongo BP, Sangeda RZ (2020). Pharmacists’ knowledge, attitude and practice regarding the dispensing of antibiotics without prescription in Tanzania: an explorative cross-sectional study. Pharmacy.

[41] Zakaa El-Din M, Samy F, Mohamed A, Hamdy F, Yasser S, Ehab M (2019). Egyptian community pharmacists' attitudes and practices towards antibiotic dispensing and antibiotic resistance; a cross-sectional survey in Greater Cairo. Curr Med Res Opin.

[42] Antimicrobial stewardship programmes in health-care facilities in low- and middle-income countries. A practical toolkit. Geneva: World Health Organization; 2019. Licence: CC BY-NC-SA 3.0 IGO. 10.1093/jacamr/dlz072 PMC8210188 34222945

[43] Afari-Asiedu S, Kinsman J, Boamah-Kaali E, Abdulai MA, Gyapong M, Sankoh O (2018). To sell or not to sell; the differences between regulatory and community demands regarding access to antibiotics in rural Ghana. J Pharm Policy Pract.

[44] Afari-Asiedu S, Oppong FB, Tostmann A, Ali Abdulai M, Boamah-Kaali E, Gyaase S (2020). Determinants of inappropriate antibiotics use in rural central ghana using a mixed methods approach. Front Public Health.

[45] Nguyen HH, Ho DP, Vu TLH, Tran KT, Tran TD, Nguyen TKC (2019). "I can make more from selling medicine when breaking the rules"—understanding the antibiotic supply network in a rural community in Viet Nam. BMC Public Health.

[46] The 2017 Amhara region population projection. Ethiopian Census—Current Year Projection—Amhara (qotera.org).

[47] Khan FU, Khan FU, Hayat K, Ahmad T, Khan A, Chang J (2021). Knowledge, attitude, and practice on antibiotics and its resistance: a two-phase mixed-methods online study among pakistani community pharmacists to promote rational antibiotic use. Int J Environ Res Public Health.

[48] Zawahir S, Lekamwasam S, Aslani P (2021). Factors related to antibiotic supply without a prescription for common infections: a cross-sectional national survey in Sri Lanka. Antibiotics (Basel).

[49] Sommanustweechai A, Chanvatik S, Sermsinsiri V, Sivilaikul S, Patcharanarumol W, Yeung S (2018). Antibiotic distribution channels in Thailand: results of key-informant interviews, reviews of drug regulations and database searches. Bull World Health Organ.

[50] Asghar S, Atif M, Mushtaq I, Malik I, Hayat K, Babar ZU (2020). Factors associated with inappropriate dispensing of antibiotics among non-pharmacist pharmacy workers. Res Social Adm Pharm.

[51] Barker AK, Brown K, Ahsan M, Sengupta S, Safdar N (2017). What drives inappropriate antibiotic dispensing? A mixed-methods study of pharmacy employee perspectives in Haryana, India. BMJ Open.

[52] Salim AM, Elgizoli B (2017). Exploring the reasons why pharmacists dispense antibiotics without prescriptions in Khartoum state. Sudan Int J Pharm Pract.

[53] Ampaire L, Muhindo A, Orikiriza P, Mwanga-Amumpaire J, Bebell L, Boum Y (2016). A review of antimicrobial resistance in East Africa. Afr J Lab Med.

[54] Mekuria B, Gebretekle GB, Bekele T, Negussie M, Kifle M, Fenta TG (2018). Bacterial resistance to fluoroquinolones and contributing factors in Addis Ababa, Ethiopia: a mixed methods study. Ethiopian Pharmaceut J.

[55] Workneh M, Katz MJ, Lamorde M, Cosgrove SE, Manabe YC (2017). Antimicrobial resistance of sterile site infections in Sub-Saharan Africa: a systematic review. Open Forum Infect Dis.

[56] Tahoon MA, Khalil MM, Hammad E, Morad WS, Ezzat S (2020). The effect of educational intervention on healthcare providers’ knowledge, attitude, & practice towards antimicrobial stewardship program at, National Liver Institute, Egypt. Egyptian Liver J.

[57] Apisarnthanarak A, Tunpornchai J, Tanawitt K, Mundy LM (2008). Nonjudicious dispensing of antibiotics by drug stores in Pratumthani, Thailand. Infect Control Hosp Epidemiol.

[58] Park S, Soumerai SB, Adams AS, Finkelstein JA, Jang S, Ross-Degnan D (2005). Antibiotic use following a Korean national policy to prohibit medication dispensing by physicians. Health Policy Plan.

[59] Santa-Ana-Tellez Y, Mantel-Teeuwisse AK, Dreser A, Leufkens HG, Wirtz VJ (2013). Impact of over-the-counter restrictions on antibiotic consumption in Brazil and Mexico. PLoS ONE.

[60] Wirtz VJ, Herrera-Patino JJ, Santa-Ana-Tellez Y, Dreser A, Elseviers M, Vander Stichele RH (2013). Analysing policy interventions to prohibit over-the-counter antibiotic sales in four Latin American countries. Trop Med Int Health.

[61] Hongoro C, Kumaranayake L (2000). Do they work? Regulating for-profit providers in Zimbabwe. Health Policy Plan.

[62] Filippini M, Ortiz LG, Masiero G (2013). Assessing the impact of national antibiotic campaigns in Europe. Eur J Health Econ.

[63] Kandeel A, Palms DL, Afifi S, Kandeel Y, Etman A, Hicks LA (2019). An educational intervention to promote appropriate antibiotic use for acute respiratory infections in a district in Egypt- pilot study. BMC Public Health.

[64] Sulis G, Gandra S (2021). Access to antibiotics: not a problem in some LMICs. Lancet Glob Health.

