# Supplementary material for: Community drug retail outlet staff’s knowledge, attitudes and practices towards non-prescription antibiotics use and antibiotic resistance in the Amhara region, Ethiopia with a focus on non-urban towns
Source: Antimicrob Resist Infect Control. 2022 Apr 29;11:64. doi: 10.1186/s13756-022-01102-1 (PMC9052473; doi:10.1186/s13756-022-01102-1)
Supplement: Supplementary file 1 — Additional file 1: Data collection instrument. [file 13756_2022_1102_MOESM1_ESM.pdf]

Additional File 1: Data collection instrument

Community drug retail outlet staff's knowledge, attitudes and practices towards non-prescription antibiotics use and antibiotic resistance in the Amhara region, Ethiopia with a focus on non-urban towns.

Sewunet Admasu Belachew[1, 2*], Lisa Hall[1], Linda A Selvey[1]

[1]School of Public Health, The University of Queensland, 288 Herston Rd, Herston, Qld 4006, Australia

[2]School of Pharmacy, Faculty of Medicine and Health Sciences, University of Gondar, Ethiopia

***Corresponding author**

Sewunet Admasu Belachew

School of Public Health, The University of Queensland, 288 Herston Rd, Herston, Qld 4006, Australia

Email: s.admasubelachew@uq.edu.au

Authors' email address

Linda A Selvey: l.selvey@uq.edu.au

Lisa Hall: l.hall3@uq.edu.au

Data collection tool for community pharmacy self-reported survey

Reference no: ____________________________ District: ______________________________

(**Please answer all questions and statements**)

Part 1: Characteristics of the community drug retail outlet (CDRO) and CDRO staff

**S.N**	**Items**	**Responses**	(√)
1	Age	…………………………………………………years	
2	Work experience (years) in CDRO		
3	Gender	Male	
		Female	
		Do not want to specify	
4	Level of education	Druggist/pharmacy technician (diploma level)	
		Pharmacist (degree level)	
		Pharmacist (master's level or more)	
		Non-pharmacy professional (health assistants, nurses etc.)	
		Non-professional	
5	CDRO employment type	CDRO owner	

		Employee	
		Other specify……………………………………………	
6	Type of CDRO	Pharmacy	
		Drug store/shop	
		Rural drug vendor	
7	Employment status	Full time	
		Part time	
8	Are you currently a registered pharmacy professional (have already certificate)?	Yes	
		No	
9	Location of the CDRO	Near to health facility (within 1km, approximately)	
		Far from the health facility (more than 1 km, approximately)	
10	Location of the CDRO	At the centre of the town (within 1km, approximately)	
		Far from the centre of the town (more than 1 km, approximately)	
11	CDRO type	Chain	
		Individual	
12	In total, how many CDRO staff work in your CDRO?		
13	How many of the CDRO staff are on duty in a single day?		
14	On average, how many clients visit your CDRO in a day?		
15	On average in a day, how many clients approach your CDRO (without prescription) requesting for management of their minor ailments (in percentage)		
16	On average, how many prescriptions do you receive per day?		
17	On average, out of the prescription received in a day, how many of them are calling for antibiotic/s (in percentage)?		
18	On average, how many of the antibiotic/s requests in a day are without prescription (in percentage)?		
19	On average, out of the non-prescription antibiotic/s request/s in a day, for how many of the requests do you supply (in percentage)?		
20	Have you received previous training on medication use and dispensing practice (with an emphasis on antimicrobials) in the last 3 years?	Yes	
		No	

Part 2: Knowledge

This section is about your knowledge of antibiotics. Please tick the relevant box in response to the statements.

For each statement you need to tick relevant one box only

S.N	Statement	Yes	No	Unsure
1	An antibiotic is a medicine used to kill or inhibit the growth of bacteria, fungus, virus, and parasites.			
2	Diphenhydramine is an antibiotic used in treating upper respiratory tract infections.			
3	Patients allergic to amoxicillin (anaphylaxis type) should not use cephalexin.			
4	An antibiotic is any agent used to kill or inhibit the growth of bacteria.			

5	If taken too often without a clear indication, antibiotics are less likely to work in the future.			
6	Diseases with viral causes can be treated with antibiotics.			
7	Antibiotics can be used as a preventive measure to fight against future microbial (i.e. fungus, bacteria, virus and parasite) attacks.			
8	Keeping leftover antibiotics from a previous course to use next time for the same infection is a good practice			
9	Patients can stop taking antibiotics when their symptoms improve before completing their prescribed course of therapy.			
10	Acute sore throat can be treated with antibiotics irrespective of the cause			
11	Fever can be treated directly with antibiotics.			
12	Bacterial infections can be treated with antibiotics			
13	Common cold and cough should always be treated with antibiotics.			
14	Wound infection can be treated with antibiotics irrespective of the cause			
15	Acute diarrhoea can be treated with antibiotics irrespective of the cause.			
16	Urinary tract infections can be treated with antibiotics irrespective of the cause.			
17	Dispensing antibiotics without a prescription contributes to inappropriate use of antibiotics			
18	Inappropriate use of antibiotics can lead to ineffective treatment.			
19	Inappropriate use of antibiotics increases the emergence of bacterial resistance to antibiotics			
20	Clients' self-medication with antibiotics contributes to the development of antibiotic resistance.			
21	Antibiotic resistance is an important and serious public health issue facing the world.			
22	Dispensing antibiotics without a prescription will lead to development of antibiotic resistance.			
23	Skipping one or two doses of an antibiotic does not contribute to the development of antibiotic resistance.			
24	Resistant bacteria cannot be spread in healthcare institutions and communities.			
25	Lack of patient adherence to prescribed antibiotic treatment regimens is one of the main causes of antibiotic resistance.			
26	In complete antibiotic course is one of the causes of antibiotic resistance.			
27	Use of antibiotics for longer than normal prescribed course is one of the causes of antibiotic resistance.			
28	The occurrence of antibiotic resistance is mainly a problem in hospital settings compared to the community			
29	Dispensing antibiotic shorter than normal course by CDRO staff is one of the main causes of antibiotic resistance.			
30	I have a clear understanding of the rules and regulations about dispensing antibiotics (with or without a prescription) in Ethiopia			
31	In Ethiopia, antibiotics are categorised under prescription only medications.			
32	CDRO staff can legally dispense antibiotics without a prescription in Ethiopia.			
33	CDRO staff can be penalised for dispensing antibiotics without a prescription.			

Part 3: Attitudes

This section is about your attitude towards antibiotics and/or antibiotic dispensing practice. Please tick the relevant box in response to the statements.

For each statement you need to tick relevant one box only

S.N	Statement	Strongly disagree	Disagree	Never agree nor disagree	Agree	Strongly agree
1	I believe antibiotics may have a role in recovering from the following health conditions, irrespective of their cause;					
	1. Fever					
	2. Common cold and cough					

	3. Acute sore throat<br>4. Acute diarrhoea<br>5. Wound infection<br>6. Uncomplicated urinary tract infection					
2	The CDRO staff should dispense antibiotics without prescription because refusing to dispense antibiotics without a prescription will negatively affect the CDRO's sales and profits.					
3	The education I acquired or the knowledge I have about antibiotics is adequate to allow me to dispense antibiotics without a prescription.					
4	The CDRO staff should dispense antibiotics without prescription because refusing clients' request to dispense antibiotics without prescription may cause client dissatisfaction with the CDRO service.					
5	The CDRO staff should dispense antibiotics without prescription as clients may easily obtain a prescription from their doctor					
6	As the pressure from the CDRO owner to increase sales and profits is high, the CDRO staff should dispense antibiotics without a prescription					
7	A CDRO staff should receive more education about antibiotics to improve appropriate dispensing of antibiotics.					
8	The CDRO staff should dispense antibiotics without prescription as they will get it easily from another retail outlet anyway.					
9	If a patient with a bacterial infection visits a CDRO, the CDRO staff should dispense antibiotics without asking for a prescription.					
10	The CDRO staff should not dispense antibiotics without a prescription because provision of antibiotics without prescription leads to the development of resistance.					
11	The CDRO staff can dispense antibiotics without a prescription because in my opinion, antibiotics use doesn't always cause resistance.					
12	If CDRO staff know that clients have neither the time nor the finance to visit a doctor, they should dispense antibiotics without the prescription.					
13	Antibiotics are already overused by the general public in Ethiopia; therefore, dispensing antibiotics without a prescription is not going to make matters worse.					
14	Dispensing antibiotics without a prescription should be more closely controlled by the authorities.					
15	A CDRO staff should encourage patients to consult a physician and get a prescription before visiting the CDRO.					
16	Promoting appropriate use of antibiotics is a shared responsibility of healthcare professionals, patients and policy makers.					
17	A CDRO staff should dispense antibiotics without prescription because it is always difficult for CDRO staff to refuse supplying					

	antibiotics without prescription with an ever increasing demand from clients.					
18	I believe that as a CDRO staff, I have the right to contribute towards the appropriate use antibiotic in Ethiopia.					
19	Clients should stop self-medicating with antibiotics to prevent the occurrence of antibiotic resistance.					
20	Dispensing antibiotics without a prescription is not a problem because;					
	1. I believe that antibiotics are safe medicines.					
	2. I believe that antibiotics have no or few side effects.					
	3. I have the ability to assess the patients' need for antibiotics.					
	4. I know whether to dispense antibiotics to the patient or refer them to a doctor.					
	5. I can properly counsel patients on appropriate use of antibiotics.					

Part 4: Practices

This section is about antibiotic/s dispensing practice. Please tell us how frequently you perform the followings, by ticking the relevant box below for each statement.

For each statement you need to tick one box only

S.N	Items	Never (0%)	Some of the time (25%)	Half of the time (50%)	Most of the time (75%)	Always 100%)
1	I dispense antibiotic/s without prescription.					
2	I dispense antibiotics without a prescription up on direct request from a client.					
3	For adult with bacterial infection, I provide antibiotics without asking for a prescription.					
4	I dispense antibiotics without prescription for a family member or someone I know closely					
5	For patients with minor ailments, I dispense antibiotics as over the counter drug.					
6	I dispense antibiotics without prescription for children with bacterial infection.					
7	For a child with viral infection, I dispense antibiotics without prescription.					
8	I dispense antibiotics without a prescription for an adult with viral infection.					
9	Have you dispensed antibiotic/s without prescription for the following condition in the last week, in what level?					

	Common cold or cough					
	Acute diarrhoea					
	Uncomplicated urinary tract infection					
	Wound infection					
	Acute sore throat					
10	In what level, do you currently dispense antibiotic/s without prescription if you encounter a patient with;					
	Common cold or cough					
	Acute diarrhoea					
	Uncomplicated urinary tract infection					
	Wound infection					
	Acute sore throat					

11. Could you please list the most common antibiotic/s dispensed without prescription in your CDRO in the space provided below?

12. Could you please tell us the main reason/s or health conditions for which antibiotic/s dispensed without prescription in your CDRO?

Thank you for your participation!

<u>መጠይቅ</u>

ማጣቀሻ ቁጥር______________________ መርጃዉ የተሰበሰበበት ወረዳ__________________________

(እባክዎ ለሁሉም ጥያቄዎች ምላሺዎን ይስጡ)

ክፍል 1: የመድሃኒት ቤቱንና የመድሃኒት ቤቱ ሰራተኞችን/ባለሙያዎችን ባህሪያት በተመለከተ የሚዳስሱ መጠይቆች

ተ.ቁ	ጥያቄ	ምላሽ	(√)
1	እድሜ	………………………………………………….በአመት	
2	የስራ ልምድ (በአመት) በመድሃኒት ቤት ዉስጥ		
3	ጾታ	ወንድ	
		ሴት	
		መግለፅ አልፈልግም	
4	የትምህርት ደረጃ	ዲፕሎማ/ፋርማሲ ቴክኒሻን(ዲፕሎማ)	
		ፋርማሲስት (ዲግሪ)	
		ፋርማሲስት (ማስተርስ ወይም በላይ)	
		የፋርማሲ ባለሙያ ያልሆነ (የጤና ረዳት, ነርስ እና የመሳሰሉት.)	
		ባለሙያ ያልሆነ	
5	የመድሃኒት ቤት ቅጥር አይነት	የመድሃኒት ቤቱ ባለቤት	
		ተቀጣሪ	
		ከተጠቀሱት ዉጭ ካለ ይጥቀሱ……………………………………………	
6	የመድሃኒት ቤቱ አይነት	ፋርማሲ/መድሃኒት ቤት/	
		መድሃኒት መደብር	
		የገጠር መድሃኒት ቤት	
7	የቅጥር ሁኔታ	ሙሉ ሰአት	
		በከፊል	
8	የፋርማሲ ሙያ ፍቃድ ምዝገባ ወረቅት አለዎት?	አዎ	
		አይ	
9	የመድሃኒት ቤቱ አድራሻ	ከጤና አገልግሎት መስጫ አጠገብ (በግምት በ 1ኪ/ሜ ክልል ዉስጥ)	
		ከጤና አገልግሎት መስጫ የራቀ (በግምት ከ 1ኪ/ሜ በላይ)	
10	የመድሃኒት ቤቱ አድራሻ	ከአገሩ እምብርት ዉስጥ (በግምት በ 1ኪ/ሜ ክልል ዉስጥ)	
		ከአገሩ እምብርት የራቀ (በግምት ከ 1ኪ/ሜ በላይ)	
11	መድሃኒት ቤትዎ ባለ ቅርንጫፍ ነዉ	አዎ	
		አይ	
12	በ አጠቃላይ፣ በመድሃኒት ቤትዎ ስንት ባለሙያ/ሰራተኛ አለ?		
13	በእያንዳንዱ ቀን ስንት ባለሙያ/ሰራተኛ በስራ ላይ ናቸዉ?		
14	በአማካኝ በቀን ስንት ህሙማን/ደንበኛ/ መድሃኒት ቤትዎን ይጎበኛል?		
15	በአማካኝ በቀን ስንት ደንበኞች ለጥቃቅን በሽታቸው (ማይነር ኤልመንት) የመድሃኒት ማዘዣ ሳይዙ ህክምና ይጠይቃሉ? በመቶኛ ይመልሱ		
16	በአማካኝ በቀን ስንት የመድሃኒት ማዘዣ ያስተናግዳሉ/ይቀበላሉ?		
17	በአማካኝ በቀን ከተቀበሉት የመድሃኒት ማዘዣ ምን ያህሉ የፀረ-ባክቴሪያ/ዎች ትዕዛዝ ነው ወይንም ፀረ-ባክቴሪያ/ዎችን አካቷል? በመቶኛ ይመልሱ		

18	በአማካኝ በቀን ከሚመጡ የፀረ-ባክቴሪያ መጠይቆች ምን ያህሉ ያለ መድሃኒት ማዘዣ ነዉ? በመቶኛ ይመልሱ		
19	በአማካኝ በቀን ያለ መድሃኒት ማዘዣ ከሚመጡ የፀረ-ባክቴሪያ/ዎች መጠይቆች ምን ያህሉን ያድላሉ (ይሸጣሉ)? በ መቶኛ ይመልሱ		
20	የመድሃኒት (በተለይ ስለፀረ-ረቂቅ ትህዋሲያን) አጠቃቀም እና እደላ ትግበራን በተመለከተ ስልጠና ወስደዉ ያዉቃሉ ባለፉት ሶስት አመታት ዉስጥ?	አዎ	
		አይ	

ክፍል 2: እዉቀትን በተመለከተ

ይህ የመጠይቅ ክፍል ስለ ፀረ-ባክቴሪያ/ዎች ስላለዎት እዉቀት ነዉ። እባክዎ ተገቢዉ ሳጥን ላይ ምልክት በማድረግ ይምረጡ።

ለእያንዳንዱ ጥያቄ አንድ ሳጥን ብቻ ላይ ነዉ ምልክት የሚያደርጉት

ተ.ቁ	ጥያቄ	አዎ	አይ	እርግጠኛ አይደለሁም
1	ፀረ-ባክቴሪያ፤ ባክቴሪያን፣ ቫይረስን፣ ፈንገስን፣ ፓራሳይትን ለመግደል ወይንም እድገታቸዉን ለመግታት የሚጠቅም መድሃኒት ነዉ።			
2	ዳይፌንሃይዳራሚን ፀረ-ባክቴሪያ ሲሆን የላይኛዉ መተንፈሻ አካላት በሽታን(ኢንፌክሽን) ለማከም ነዉ የምንጠቀምበት።			
3	ለአሞክሳሲሊን አለርጂ (የሰዉነት አለመቀበል ካጋጠመዎ) ከሆኑ ሴፋሌክዚን መጠቀም የለባቸዉም።			
4	ፀረ-ባክቴሪያ ባክቴሪያን ለመግደል ወይም እድገታቸዉን ለመግታት የሚያገለግል መድሃኒት ነዉ።			
5	ያለ ግልፅ ትዕዛዝ ፀረ-ባክቴሪያ/ዎች ዘወትር ከተወሰዱ፤ ወደ ፊት የመስራታቸዉ ነገር ዝቅተኛ ነዉ።			
6	ፀረ-ባክቴሪያ ወደ ፊት የሚያጋጥሙ ረቂቅ-ተህዋስያንን (ባክቴሪያን፣ ቫይረስን፣ ፈንገስን፣ ፓራሳይትን) ለመዋጋት እንደ ቅድመ መከላከያ ዘዴ መጠቀም እንችላለን።			
7	ከበፊት ህክምና የተረፈ ፀረ-ባክቴሪያ አስቀምጦ በቀጣይ ለተመሳሳይ ተላላፊ በሽታ(ኢንፌክሽን) መጠቀም ጥሩ ተግባር (ልምድ) ነዉ።			
8	ህሙማን የበሽታቸዉ ምልክት/ስሜት ሲሻሻል ፀረ-ባክቴሪያዉን የታዘዘላቸዉ ግዜ ባያልቅም መዉሰድ ማቆም ይችላሉ			
9	በቫይረስ ምክንያት የሚመጡ በሽታወች በፀረ-ባክቴሪያ መታከም ይችላሉ።			
10	አጣዳፊ በጉንፋን የተዘጋ ጉሮሮን ለማከም መንስኤዉ ምንም ይሁን ፀረ-ባክቴሪያ መጠቀም እንችላለን			
11	የሰዉነት ትኩሳትን ቀጥታ በፀረ-ባክቴሪያ ማከም እንችላለን።			
12	የ ባክቴሪያ ኢንፌክሽንን (በሽታን) በፀረ-ባክቴሪያ ማከም ይቻላል።			
13	ጉንፋን እና ሳል ምንግዜም ዘወትር መታከም ያለባቸዉ በፀረ-ባክቴሪያ ነዉ።			
14	የቁስል ኢንፌክሽንን መንስኤዉ ምንም ይሁን በፀረ-ባክቴሪያ ማከም ይቻላል።			
15	አጣዳፊ ተቅማጥን መንስኤዉ ምንም ይሁን በፀረ-ባክቴሪያ ማከም ይቻላል።			
16	የሽንት ቧንቧ ኢንፌክሽንን መንስኤዉ ምንም ይሁን በፀረ-ባክቴሪያ ማከም ይቻላል።			
17	ያለ መድሃኒት ማዘዣ ፀረ-ባክቴሪያን ማደል በህሙማን ዘንድ ትክክል ላልሆነ የፀረ-ባክቴሪያ አጠቃቀም አስተዋፆ አለዉ።			
18	ትክክል ያልሆነ የፀረ-ባክቴሪያ አጠቃቀም ህክምናዉን(መድሃኒቱን) ጥቅም የለሽ ያደርገዋል።			
19	ትክክል ያልሆነ የፀረ-ባክቴሪያ/ዎች አጠቃቀም የፀረ-ባክቴሪያ መቋቋም ክስተትን ይጨምራል።			
20	የደንበኞች በፀረ-ባክቴሪያ ራስን ማከም ፀረ-ባክቴሪያ የመቋቋም እድገት ላይ አስተዋጽዖ አለዉ።			
21	የፀረ-ባክቴሪያ መቋቋም አለማችን የተጋረጠባት አስፈላጊ እና አንገብጋቢ የማህበረሰብ ጤና ጉዳይ ነዉ።.			
22	ያለ መድሃኒት ማዘዣ ፀረ-ባክቴሪያን ማደል የፀረ-ባክቴሪያ መቋቋም እንዲያድግ (እንዲከሰት) ያደርጋል።			
23	አንድ ወይም ሁለት የፀረ-ባክቴሪያን መጠን (ዶዝ) መዝለል የፀረ-ባክቴሪያ መቋቋም እድገት (ክስተት) ላይ አስተዋፆ የለዉም።			
24	ፀረ-ባክቴሪያን መቋቋም የሚችሉ ባክቴሪያወች በጤና ተቋም ሆነ በህብረተሰቡ ዉስጥ አይስፋፉም (አይዛመቱም)።			
25	በመድሃኒት ማዘዣ የታዘዙ የፀረ-ባክቴሪያ መድሃኒትን ተከታትሎ ያለመዉሰድ ችግር ከዋና ዋናወቹ የፀረ-ባክቴሪያ መቋቋም መንሰኤዎች አንዱ ነዉ።			
26	ፀረ-ባክቴሪያ በከፊል ብቻ ወሰዶ ማቆም አንዱ ለፀረ-ባክቴሪያ መቋቋም መንሰኤ ነዉ።			
27	በመድሃኒት ማዘዣ ከታዘዘዉ ትክክል ጊዜ በላይ ፀረ-ባክቴሪያን መዉሰድ አንዱ የፀረ-ባክቴሪያ መቋቋም መንሰኤ ነዉ።			

28	የፀረ-ባክቴሪያ መቋቋም ችግር በአብዛኛዉ የሚከሰተዉ በጤና ተቋማት(ከሆስፒታል) ነዉ ከህብረተሰቡ ዉስጥ ከሚከሰተዉ ጋር ሲነፃፀር።			
29	የመድሃኒት ቤት ሰራተኞች/ባለሙያዎች ፀረ-ባክቴሪያን ከትክክለኛው ግዜ ያጠረ አድርጎ ማደል ከዋና ዋናወቹ የፀረ-ባክቴሪያ መቋቋም መንስኤዎች አንዱ ነዉ።			
30	እኔ በኢትዮጵያ ዉስጥ የፀረ-ባክቴሪያ እደላን (በመድሃኒት ማዘዣ ወይንም ያለማዘዣ) በተመለከተ ስላሉ ደንቦችና መመሪያወች ግልፅ የሆነ ግንዛቤ አለኝ።			
31	በኢትዮጵያዊ ዉስጥ ፀረ-ባክቴሪያ የሚካተቱት በመድሃኒት ማዘዣ ብቻ ከሚሰጡ መድሃኒቶች ዉስጥ ነዉ።			
32	በኢትዮጵያዊ ዉስጥ የፋርማሲ ባለሙያዎች/ሰራተኞች ያለመድሃኒት ማዘዣ ፀረ-ባክቴሪያን እንዲያድሉ ህጉ ይፈቅድላቸዋል።			
33	የመድሃኒት ቤት ሰራተኞች/ባለሙያዎች ፀረ-ባክቴሪያን ያለ መድሃኒት ማዘዣ ማደል ((መስጠት) ሊያስቀጣቸዉ ይችላል።			

ክፍል 3: አመለካከትን በተመለከተ

ይህ የመጠይቅ ክፍል ስለ ፀረ-ባክቴሪያ/ዎች ስላለዎት አመለካከት ነዉ። እባክዎ ተገቢዉ ሳጥን ላይ ምልክት በማድረግ ይምረጡ።

ለእያንዳንዱ ጥያቄ አንድ ሳጥን ብቻ ላይ ነዉ ምልክት የሚያደርጉት

ተ.ቁ	ጥያቄ	በጣም አልስማማም	አልስማማም	ገለልተኛ	እስማማለሁ	በጣም እስማማለሁ
1	እንደኔ እምነት ፀረ-ባክቴሪያ/ዎች ከሚከተሉት የጤና እክሎች ለማገገም ሚና ሊኖራቸው ይችላል መንሳኤያቸው ምንም ቢሆን፤					
	7. ትኩሳት					
	8. ጉንፋን እና ሳል					
	9. አጣዳፊ የጉሮሮ ህመም					
	10. አጣዳፊ ተቅማጥ					
	11. የቁስል ኢንፌክሽን					
	12. ያልተባባሰ የሽንት ቧንቧ ኢንፌክሽን					
2	የመድሃኒት ቤት ሰራተኞች/ባለሙያዎች ፀረ-ባክቴሪያ/ዎችን ያለመድሃኒት ማዘዣ ማደል አለባቸዉ ምክንያቱም ፀረ-ባክቴሪያን ያለመድሃኒት ማዘዣ ከማደል መቆጠብ የመድሃኒት ቤት ሽያጭ እና ትርፍ ላይ አሉታዊ ትፅዕኖ ስለሚኖረው።					
3	ስለ ፀረ-ባክቴሪያ/ዎች በትምህርት ያገኘሁት ወይንም ያለኝ እዉቀት ፀረ-ባክቴሪያ/ዎችን ያለ መድሃኒት ማዘዣ ለማደል ያስችለኛል ወይንም በቂ ነዉ።					
4	የመድሃኒት ቤት ሰራተኞች/ባለሙያዎች ፀረ-ባክቴሪያ/ዎችን ያለመድሃኒት ማዘዣ ማደል አለባቸዉ ምክንያቱም ፀረ-ባክቴሪያን ያለመድሃኒት ማዘዣ ከማደል መቆጠብ ህሙማን በመድሃኒት ቤቱ አገልግሎት ላይ እርካታ እንዳይኖራቸዉ መንስኤ ይሆናል።					
5	የመድሃኒት ቤት ሰራተኞች/ባለሙያዎች ፀረ-ባክቴሪያ/ዎችን ያለመድሃኒት ማዘዣ ማደል አለባቸዉ ምክንያቱም ህሙማን ፀረ-ባክቴሪያ/ዎቹ ያለመድሃኒት ማዘዣ ካልተሸጠላቸዉ፤ ህሙማን በቀላሉ ከሃኪማቸዉ ፀረ-ባክቴሪያ የተፃፈበት የመድሃኒት ማዘዣ ማግኘት ይችላሉ።					
6	ሽያጭ እና ትርፍን ለመጨመር ከመድሃኒት ቤት ባለቤት የሚመጣ ጫና ከባድ ስለሆነ፤ የመድሃኒት ቤት ሰራተኞች/ባለሙያዎች ፀረ-ባክቴሪያ/ዎችን ያለመድሃኒት ማዘዣ ማደል አለባቸዉ።					
7	ትክክለኛ የፀረ-ባክቴሪያ እደላን ለማሻሻል በህብረተሰቡ ዉስጥ የሚገኙ መድሃኒት ቤቶች ዉስጥ የሚሰሩ የመድሃኒት ቤቶቹ ሰራተኞች/ባለሙያዎች ስለ ፀረ-ባክቴሪያ/ዎች በቂ ትምህርት ማገኘት አለባቸዉ።					

8	የመድሃኒት ቤት ሰራተኛ/ባለሙያ ፀረ-ባክቴሪያ/ዎችን ያለመድሃኒት ማዘዣ ማደል አለበት ምክንያቱም ህሙማን ፀረ-ባክቴሪያ/ዎቹ ያለመድሃኒት ማዘዣ ካልተሸጠላቸዉ፤ ፀረ-ባክቴሪያ/ዎችን በቀላሉ ከሌላ መድሃኒት ቤት ማግኘት ይችላሉ።					
9	ህሙማን ወደ መድሃኒት ቤቱ የመጡበት ምክንያት የባክቴሪያ ኢንፌክሽን ከሆነ የመድሃኒት ቤቱ ሰራተኞች/ባለሙያዎች ፀረ-ባክቴሪያ/ዎችን ያለመድሃኒት ማዘዣ ማደል ይኖርባቸዋል።					
10	የመድሃኒት ቤት ሰራተኞች/ባለሙያዎች ፀረ-ባክቴሪያ/ዎችን ያለመድሃኒት ማዘዣ ማደል የለባቸዉም ምክንያቱም ያለ መድሃኒት ማዘዣ ፀረ-ባክቴሪያዎችን ማደል የፀረ-ባክቴሪያዎችን መቋቋም እንዲከሰት ያደረጋል።					
11	የመድሃኒት ቤት ሰራተኞች/ባለሙያዎች ፀረ-ባክቴሪያ/ዎችን ያለ መድሃኒት ማዘዣ ማደል ይችላሉ ምክንያቱም እንደ እኔ አስተያየት የፀረ-ባክቴሪያ/ዎች የአጠቃቀም ሁኔታ ሁልግዜ የፀረ-ባክቴሪያዎች መቋቋም መንስኤ ስለማይሆን።					
12	የመድሃኒት ቤት ሰራተኞች/ባለሙያዎች ህሙማን ሃኪም ለመጎብኘት ሰዐትም ሆነ ገንዘብ እንደሌላቸዉ ካወቁ ፀረ-ባክቴሪያ/ዎችን ያለመድሃኒት ማዘዣ ማደል ይኖርባቸዋል።					
13	በኢትዮጵያዊ ዉስጥ በጠቅላላዉ ህብረተሰብ ዘንድ ቀድሞዎኑ ፀረ-ባክቴሪያ/ዎችን ከመጥን በላይ ተጠቅመዉባቸዋል፤ ስለዚህ ፀረ-ባክቴሪያ/ዎችን ያለ መድሃኒት ማዘዣ ማደል ነገሮችን/ችግሮችን ሊያባብስ አይችልም።					
14	ያለ መድሃኒት ማዘዣ የሚደረግን የፀረ-ባክቴሪያ/ዎችን እደላ የቁጥጥር ባለስልጣናት በቅርብ ሊቆጣጠሩት ይገባል።					
15	የመድሃኒት ቤት ሰራተኞች/ባለሙያዎች ህሙማን መድሃኒት ቤቶችን ከመጎብኘታቸዉ በፊት ሃኪምን እንዲያማክሩ እና መድሃኒት ማዘዣ እንዲያገኙ ማበረታት አለባቸው።					
16	ትክክል የሆነ የፀረ-ባክቴሪያ/ዎች አጠቃቀምን ማጎልበት የጤና ባለሙያዎች፣ ህሙማን እና ፖሊሲ ሰዎች የጋራ ድርሻ ነዉ።					
17	እንደእኔ እምነት እንደ አንድ የመድሃኒት ቤት ሰራተኛ/ባለሙያ፤ በኢትዮጵያ ዉስጥ ትክክለኛ የፀረ-ባክቴሪያ/ዎች አጠቃቀም እንዲኖር አስተዋፆ የማድረግ መብት አለኝ።					
18	የመድሃኒት ቤት ሰራተኞች/ባለሙያዎች ፀረ-ባክቴሪያ/ዎችን ያለመድሃኒት ማዘዣ ማደል አለባቸዉ ምክንያቱም የህሙማን ፊላጎት/ጥያቄ በየግዜዉ እየጨመረ ባለበት ሁኔታ ፀረ-ባክቴሪያን ያለመድሃኒት ማዘዣ ከማደል መቆጠብ ሁልግዜ አስቸጋሪ ሰለሆነ።					
19	የፀረ-ባክቴሪያ/ዎችን መቋቋም መከሰት ለመከላከል ህሙማን ራስን በራስ በፀረ-ባክቴሪያ/ዎች ማከም ማቆም አለባቸዉ።					
20	ፀረ-ባክቴሪያ/ዎችን ያለ መድሃኒት ማዘዣ ማደል ችግር የለዉም ምክንያቱም፤					
	6. እንደእኔ እምነት ፀረ-ባክቴሪያ/ዎች ምንም ችግር የማያስከትሉ መድሃኒቶች ስለሆኑ።					
	7. እንደእኔ እምነት ፀረ-ባክቴሪያ/ዎች ምንም ወይንም በጣም ትንሽ የጎንዮሽ ጉዳት ስለሚያስከትሉ።					
	8. እኔ የህሙማንን የፀረ-ባክቴሪያ ፍላጎት መርምሮ ወይንም ለይቶ የማወቅ ብቃቱ ስላለኝ።					
	9. እኔ ለህሙማን ፀረ-ባክቴሪያ/ዎችን ማዘዝ ያለብኝ እንደሆነ ወይንም ወደ ሃኪም መላክ እንዳለብኝ ስለማዉቅ።					
	10. እኔ በትክክል ህሙማንን ስለ ትክክለኛ ፀረ-ባክቴሪያ/ዎች አጠቃቀም መምከር ስለምችል።					

ክፍል 4: ተግባርን በተመለከተ

ይህ የመጠይቅ ክፍል ስለ ፀረ-ባክቴሪያ/ዎች እደላ አተገባበርን በተመለከተ ነዉ። እባክዎ ተገቢዉ ሳጥን ላይ ምልክት በማድረግ አተገባበርዎ በምን ያህል መጠን/ድግግሞሽ እንደሆነ ያሳዉቁን።

ለእያንዳንዱ ጥያቄ አንድ ሳጥን ብቻ ላይ ነዉ ምልክት የሚያደርጉት

ተ.ቁ	ጥያቄ	በፍፁም (0%)	አንዳንድ ግዜ (25%)	በከፊል (ገሚሱን ግዜ) (50%)	አብዝሃኛዉን ግዜ (75%)	ሁል ግዜ (100%)
1	እኔ ፀረ-ባክቴሪያ/ዎችን ያለ መድሃኒት ማዘዣ እሰጣለሁ።					
2	ከህሙማን/ደንበኞች በቀጥታ ጥያቄዉ ከመጣ ፀረ-ባክቴሪያ/ዎችን ያለ መድሃኒት ማዘዣ እሰጣለሁ።					
3	በባክቴሪያል ኢንፌክሽን ለተያዘ ጎልማሳ፤ የመድሃኒት ማዘዣ ሳልጠይቅ ፀረ-ባክቴሪያ እሰጣለሁ።					
4	ታማሚዉ የቤተሰብ አባል ወይንም የማዉቀው ሰዉ ከሆነ ያለመድሃኒት ማዘዣ ፀረ-ባክቴሪያ/ዎችን እሰጣለሁ።					
5	ህሙማን በጥቃቅን ህመሞች(ማይነር ኤልመንት) ከመጡ ፀረ-ባክቴሪያ/ዎችን ያለመድሃኒት ማዘዣ እንደ ሌሎች ያለማዘዣ እንደሚሰጡ መድሃኒቶች እሰጣለሁ።					
6	ፀረ-ባክቴሪያ/ዎችን ያለ መድሃኒት ማዘዣ በኢንፌክሽን ለተያዙ ልጆች እሰጣለሁ።					
7	በቫይረስ ኢንፌክሽን ለተያዘ ልጅ፤ ፀረ-ባክቴሪያ/ዎችን ያለመድሃኒት ማዘዣ እሰጣለሁ።					
8	በቫይረስ ኢንፌክሽን ለተያዘ ጎልማሳ፤ ፀረ-ባክቴሪያ/ዎችን ያለ መድሃኒት ማዘዣ እሰጣለሁ።					
9	ለሚከተሉት የጤና እክሎች በባለፈው ሳምንት ያለ መድሃኒት ማዘዣ ፀረ-ባክቴሪያ/ዎች በምን ያህል መጠን ስጥተዋል?					
	ጉንፋን ወይንም ሳል					
	አጣዳፊ ተቅማጥ					
	ያልተባባሰ የሽንት ቧንቧ ኢንፌንክሽን					
	የቁስል ኢንፌክሽን					
	አጣዳፊ የጉሮሮ ማበጥ					
10	የሚከተልኩትን በሽታዎች ወይንም የበሽታ ምልክቶችን የሚያሳዩ ህሙማን በአሁኑ ግዜ ቢመጡ ፀረ-ባክቴሪያ/ዎችን ያለ መድሃኒት ማዘዣ በምን ያህል መጠን ይሰጣሉ?					
	ጉንፋን ወይንም ሳል					
	አጣዳፊ ተቅማጥ					
	ያልተባባሰ የሽንት ቧንቧ ኢንፌክሽን					
	የቁስል ኢንፌክሽን					
	አጣዳፊ የጉሮሮ ማበጥ					

11. በአብዛሃኛው በመድሃኒት ቤትዎ ያለ መድሃኒት ማዘዣ የሚሰጡ ፀረ-ባክቴሪያ/ዎችን እባክዎ ከታች ባለው ክፍት ቦታ ይዘርዝሩልን?

12. በመድሃኒት ቤትዎ ፀረ-ባክቴሪያ/ዎችን ያለ መድሃኒት ማዘዣ በአብዛሃኛዉ የሚሰጡበት ምክንያት/ቶችን ወይንም የጤና እክሎችን እባክዎ ይንገሩን?

ስለተሳተፋችሁ እናመሰግናለን!
