# Supplementary material for: Community drug retail outlet staff’s knowledge, attitudes and practices towards non-prescription antibiotics use and antibiotic resistance in the Amhara region, Ethiopia with a focus on non-urban towns
Source: Antimicrob Resist Infect Control. 2022 Apr 29;11:64. doi: 10.1186/s13756-022-01102-1 (PMC9052473; doi:10.1186/s13756-022-01102-1)
Supplement: Supplementary file 2 — Additional file 2: Fig. S1. The types of antibiotics most dispensed without prescription as reported by the 153 participants. [file 13756_2022_1102_MOESM2_ESM.pdf]

Additional file 2: Figure S1

Community drug retail outlet staff's knowledge, attitudes and practices towards non-prescription antibiotics use and antibiotic resistance in the Amhara region, Ethiopia with a focus on non-urban towns.

Sewunet Admasu Belachew[1, 2*], Lisa Hall[1], Linda A Selvey[1]

[1]School of Public Health, The University of Queensland, 288 Herston Rd, Herston, Qld 4006, Australia

[2]School of Pharmacy, Faculty of Medicine and Health Sciences, University of Gondar, Ethiopia

***Corresponding author**

Sewunet Admasu Belachew

School of Public Health, The University of Queensland, 288 Herston Rd, Herston, Qld 4006, Australia

Email: s.admasubelachew@uq.edu.au

Authors' email address

Linda A Selvey: l.selvey@uq.edu.au

Lisa Hall: l.hall3@uq.edu.au

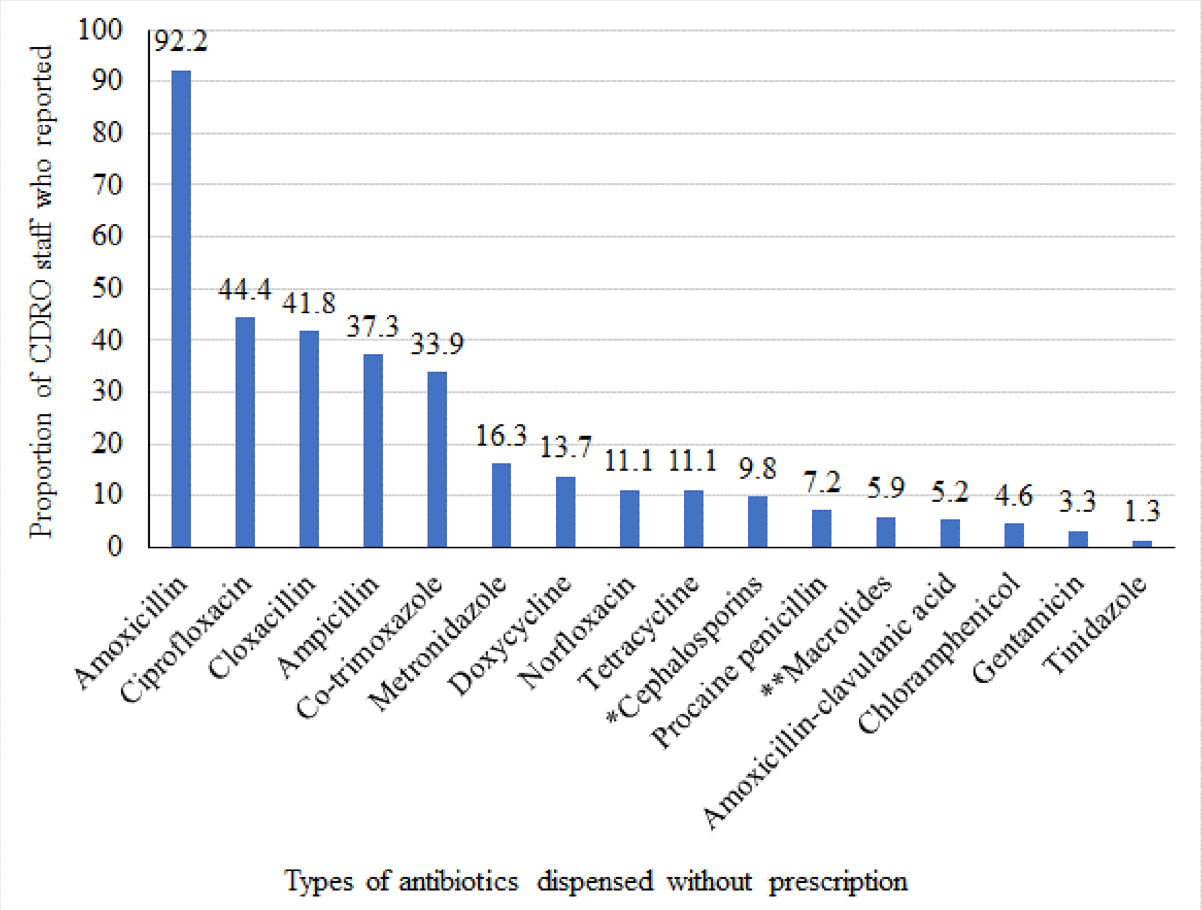

Fig. S 1: The types of antibiotics most dispensed without prescription as reported by the 153 participants

***Cephalosporins**: cephalexin and/or ceftriaxone and/or cefixime

****Macrolides:** azithromycin and/or erythromycin
